# An insight into the functional alterations in the gut microbiome of healthy adults in response to a multi-strain probiotic intake: a single arm open label trial

**DOI:** 10.3389/fcimb.2023.1240267

**Published:** 2023-09-29

**Authors:** Andrea Rodenes-Gavidia, Araceli Lamelas, Sarah Bloor, Anthony Hobson, Sam Treadway, Jordan Haworth, Vineetha Vijayakumar, Malwina Naghibi, Richard Day, Empar Chenoll

**Affiliations:** ^1^ ADM BIOPOLIS, University of Valencia Science Park (Parc Científic de la Universitat de València), Valencia, Spain; ^2^ Functional Gut Clinic, Manchester, United Kingdom; ^3^ Anglia Ruskin University, Essex, Norwich, United Kingdom; ^4^ Medical Department, ADM Health & Wellness, Somerset, United Kingdom

**Keywords:** gut microbiome, metagenomics, microbiome, probiotics, healthy adults

## Abstract

**Background:**

Probiotic supplements, by definition, provide a benefit to the host, but few studies have investigated the effect of probiotic supplements in healthy adult populations.

**Purpose:**

The present, single arm, open label clinical trial, evaluated compositional and functional changes in the fecal microbiome of healthy adults after supplementation with a 14-strain probiotic.

**Methods:**

We analysed the effect of a 14-strain probiotic blend (*Bacillus subtilis* NCIMB 30223, *Bifidobacterium bifidum* NCIMB 30179, *B. breve* NCIMB 30180, *B. infantis* NCIMB 30181, *B. longum* NCIMB 30182, *Lactobacillus helveticus* NCIMB 30184, *L. delbrueckii* subsp. *bulgaricus* NCIMB 30186, *Lacticaseibacillus paracasei* NCIMB 30185, *Lactiplantibacillus plantarum* NCIMB 30187, *Lacticaseibacillus rhamnosus* NCIMB 30188, *L. helveticus* NCIMB 30224, *Lactobacillus salivarius* NCIMB 30225, *Lactococcus lacti*s subsp. *lactis* NCIMB 30222, and *Streptococcus thermophilus* NCIMB 30189), on the faecal microbiota of healthy young adults (n=41) in a single arm study. The adults consumed 4 capsules daily of the 14 strain blend(8 billion colony forming units/day) for 8 weeks. Compositional and functional changes in faecal microbiota before and after supplementation were assessed using shotgun metagenomic sequencing. Fasting breath analysis, faecal biochemistry and bowel habits were also assessed.

**Results:**

In healthy adult participants, no significant changes to the overall alpha- or beta-diversity was observed after 8 weeks of multi-strain probiotic supplementation. However, in a simplified model that considered only time and individual differences, significant decreases (p < 0.05) in family *Odoribacteraceae* and *Bacteroidaceae* abundance and a significant increase (p < 0.05) in genus *Megamonas* abundance were observed. At a functional level, there were significant changes in functional gene abundance related to several functional pathways, including phenylalanine metabolism, O-antigen nucleotide sugar biosynthesis, bacterial chemotaxis, and flagellar assembly. No significant changes in stool form or frequency, fecal biochemistry, or methane and hydrogen breath tests were observed.

**Conclusion:**

In healthy young adults, overall alpha- and beta-diversity did not change in response to probiotic intake even though modest compositional changes at the family and genus level were observed. However, at functional level, results identified changes in gene abundance for several functional pathways.

## 1 Introduction

The human gut microbiota is a key mediator of host health and is known to affect many physiological processes, such as digestion, metabolism, immune function and inhibition of pathogen colonization (Endt et al., 2010; Belizário and Faintuch, 2018; Koradia et al., 2019; Lee and Chang, 2021; Vos et al., 2022). Collectively, the microbes that constitute the gut microbiome number in their trillions, with the overall relative abundance of specific populations varying considerably between individuals (Rinninella et al., 2019).

The gut microbiome can be impacted by many extrinsic factors including diet, stress, immunosuppressants, antibiotics, surgery, and radiotherapy, resulting in changes in bacterial abundance and diversity (Belizário and Faintuch, 2018). Studies assessing the compositional diversity and functional capacity of gut microbiota have highlighted several negative health outcomes associated with microbiome perturbations, including certain cancers, type 2 diabetes and obesity (Qin et al., 2012; Feng et al., 2015; Forslund et al., 2015; Yu et al., 2017). As such, interventions that positively impact the gut microbiome that are safe and effective are widely considered valuable to promoting good health (Williams, 2010).

Probiotics are defined as ‘non-pathogenic live micro-organisms that, when administered in adequate amounts, confer a health benefit to the host’ (Hill et al., 2014). Several benefits of probiotics to areas of human health have been reported, including allergies, gastrointestinal (GI) distress, support for immune function, and support of intestinal microbiome homeostasis (Floch, 2014; Arora and Baldi, 2015; Currò et al., 2017). Lactobacilli and bifidobacteria are the most commonly used micro-organisms in probiotic products, and several studies have reported that these probiotics are able to modulate intestinal microbiota composition and exert immunomodulatory activities, resulting in improvement to human health, in addition to inhibiting the adhesion of pathogenic gram-negative bacteria in the intestinal environment (Servin, 2004; Markowiak and Śliżewska, 2017; Li et al., 2019; Averina et al., 2021).

The effectiveness of the 14 strain probiotic blend has been assessed in several randomized, placebo-controlled trials (RCTs). The 14 strain blend was reported to be safe and efficacious, improving various aspects of GI distress over a period of 4 months in irritable bowel syndrome (IBS) diarrhea-predominant subjects (Ishaque et al., 2018). Furthermore, the multi-strain probiotic significantly reduced the frequency and severity of migraines in subjects experiencing episodic and chronic migraines (Martami et al., 2019) and significantly improved depression scores in individuals with low mood (Baião et al., 2022).

Yet, among these positive results, there are no data evaluating the effects of the 14 strain blend on the fecal microbiome in healthy individuals. While there is no scientific consensus on what constitutes a “healthy” microbiome, healthy populations are known to present variations in microbiota patterns. These variations have been attributed to a wide variety of factors, including age, diet, lifestyle habits, cohabitants, and many others, however research demonstrating how probiotic supplements interact with and modulate the gut microbiota and exert positive effects on healthy adults remains limited (Scepanovic et al., 2019).

Historically, most research has focused on understanding the effect of probiotics on compositional changes to the microbiota and relatively little research has focused on functional changes. Compositional outcomes include alpha diversity, beta diversity, differential abundance across taxonomic levels whereas functional alterations measure actual (metagenomic) differential abundance of microbial genes. Therefore, the primary focus of this study was to assess both compositional and functional changes in the microbiome of healthy individuals using shotgun metagenomics following 8-weeks of daily multi-strain probiotic intake. Additional measurements included examining correlations between microbiota and hydrogen and methane production from breath samples, determination of changes in stool frequency and consistency, and analysis of fecal biochemistry.

## 2 Materials and methods

### 2.1 Compliance with ethical standards

The study was conducted according to the guidelines in the Declaration of Helsinki. The protocol was reviewed and approved by the NHS Research Ethics Committee (REC reference 19/SW/0203). Written informed consent was obtained from all participants prior to any study‐related procedures being performed. The study was registered at www.ClinicalTrials.gov (NCT04554641).

### 2.2 Intervention

A multi-strain probiotic supplement containing 14 live bacterial strains (*Bacillus subtilis* NCIMB 30223, *Bifidobacterium bifidum* NCIMB 30179, *B. breve* NCIMB 30180, *B. infantis* NCIMB 30181, *B. longum* NCIMB 30182, *Lactobacillus helveticus* NCIMB 30184, *L. delbrueckii* subsp*. bulgaricus* NCIMB 30186, *Lacticaseibacillus paracasei* NCIMB 30185, *Lactiplantibacillus plantarum* NCIMB 30187, *Lacticaseibacillus rhamnosus* NCIMB 30188, *L. helveticus* NCIMB 30224, *Lactobacillus salivarius* NCIMB 30225, *Lactococcus lactis* subsp*. lactis* NCIMB 30222, and *Streptococcus thermophilus* NCIMB 30189).

The multi-strain probiotic was supplied by ADM Protexin as capsules that were administered orally. All participants consumed 4 capsules daily (2 pre‐morning meal and 2 pre‐evening meal) for 8 weeks (56 days [ ± 2 days]), equating to a total daily intake of 8 billion colony forming units (CFUs) per day.

### 2.3 Study participants

Participants were recruited through advertisements on several social media platforms within the UK. After providing written informed consent, participants completed a screening questionnaire.

The study enrolled a total of 41 healthy adult males and females between 18 to 40 years old with a body mass index (BMI) between 18.5 to 30.0 kg/m^2^. Enrolled participants were not taking regular prescription medicines and had no selective/restricted diets or used diet replacements (e.g., vegan or Huel). Participants were excluded if they had any significant medical condition, prior abdominal surgery, a significant history of migraines (≥5 prior attacks), were taking ongoing therapy with a medication known to affect the gut microbiome (including the use of antibiotics, proton pump inhibitors, or antidepressants within the 8 weeks prior to enrollment or regular use of laxatives or anti-diarrheal medications), consumed >14 units alcohol/week, were pregnant or breastfeeding, regularly consumed probiotics, prebiotics, or fiber supplements, or consumed any probiotics in the 2 months prior to enrolment. Participants also had to be willing to exclude other probiotic products from their diet during the study period.

### 2.4 Study design

At the baseline visit (Day 0), height and weight were measured, and BMI was calculated. Participants were given a study diary and instructed on how to complete it daily. The study diary recorded information about the number of bowel movements per day, stool consistency, and experiences of abdominal pain, bloating, and flatulence.

**Figure 1:**
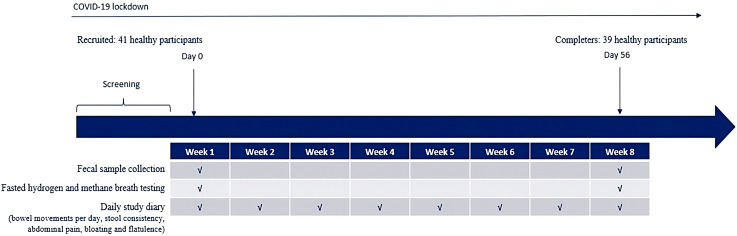
Study design schematic.

Additional samples collected at baseline included hydrogen and methane breath testing, which was performed after a fasting period of 12 hours, during which only water was permitted. A baseline stool sample was collected for fecal microbiome analysis. At the end of the study, hydrogen and methane breath testing were performed in the fasted state, and an additional stool sample was collected for fecal microbiome analysis. All baseline samples were compared to those collected at the end of study visit (Day 56 [ ± 2 days]). A schema of the overall study design is detailed in **Figure 1**.

### 2.5 Stool frequency and consistency

Based on study diary data, differences in stool frequency and consistency from baseline to the end of the study were determined using a paired t-test and Chi-square, respectively. Statistical significance was defined by a P-value < 0.05.

### 2.6 Hydrogen and methane breath sampling

Breath samples were provided via 3 to 5 second exhalation at both baseline (Day 0) and at the end of study visit (Day 56 [ ± 2 days]) after the 12-hour fasting period. Samples were analyzed using gas chromatography performed on an Agilent 7890B (Agilent, Santa Clara, United States). Carbon dioxide (CO_2_) was used as a correction factor according to the method outlined by Rezaie et al. (Rezaie et al., 2017). A threshold of ≥10 ppm of methane was used to establish whether an individual was a methane producer (Rezaie et al., 2017).

### 2.7 Fecal sample collection and microbiome analysis

#### 2.7.1 Fecal sample collection and DNA extraction

Stool samples were collected at baseline (Day 0) and at the end of study visit (Day 56 [ ± 2 days]) for microbiome analysis. Stool samples were frozen at -20°C on the day of collection and stored at this temperature until processing. Prior to the COVID-19 pandemic, study participants would deposit any samples at the study site on the day of collection for immediate freezing. During any COVID-19 lockdown, to maintain cold chain logistics, collected samples were frozen at home until the study team could safely collect the samples. Samples were then shipped to Genewiz facilities (Genewiz UK Ltd., Essex, UK) where they were stored at -80°C until further analyses were performed. Fecal DNA was extracted by mechanical disruption using FastPrep‐24™ instrument (MP Biomedicals. Following this, DNA was isolated using QIAamp^®^PowerFecal^®^Pro DNA-kit (QIAGEN^®^, Hilden, Germany). Finally, DNA preparation was subjected to quality control by spectrophotometry on a NanoDrop™ 2000c spectrophotometer (Thermo Fisher Scientific, Waltham, United States).

#### 2.7.2 Fecal DNA metagenomic shotgun sequencing

Fecal DNA was quantified using a Qubit Fluorometer (Themo Fisher Scientific, Carlsbad, United States). Sequencing libraries were prepared with Nextera XT Library kit (Illumina, San Diego, United States) following the manufacturer’s instructions. The samples were sequenced using a NovaSeq 6000 platform with paired-end reads of 151 base pairs, resulting in 45.2 million ( ± 2.8 million) paired-ends per sample. Demultiplexed reads were filtered with BBTools suite and quality filtering was performed with BBMap (version 38.36) (Bushnell et al., 2017). Reads were filtered for 97% identity to the human genome (hg19) using NGLess version 1.0.0‐Linux64 (Coelho et al., 2019). The remaining ‘high-quality sequences’ were used for bioinformatic analysis (36.1 million ± 3.3 million paired-end reads per sample).

#### 2.7.3 Fecal DNA bioinformatic analysis

Metaphlan (version 4) (Blanco-Míguez et al., 2023) was used to assign the taxonomy to the reads. The ‘high‐quality sequences’ were assembled using MEGAHIT genome assembler (version 3.13.0) (Li et al., 2015). Genes in contigs >500 bps were predicted using Prodigal (version 2.6.3) (Hyatt et al., 2010). Kyoto Encyclopedia of Genes and Genomes (KEGG) functional annotation was performed using web server GhostKoala (Kanehisa et al., 2016). NGLess version 1.0.0‐Linux64 (Coelho et al., 2019) was used to map the ‘high‐quality sequences’ to the predicted genes, generating a matrix with the gene counts for each sample.

For biomarker identification, the feature table using calcNormFactors function was normalized, the TMMwsp option. The limma R package (version 3.42.2) function voom (Law et al., 2014) was used to convert normalized counts to log2‐counts‐per million, and precision weights were assigned to each observation based on the mean‐variance trend. Weighted linear regression models were fitted using the lmFit, eBayes, and topTable functions in the limma package. Empirical Bayes moderated t-statistics were calculated, and Benjamini-Hochberg false discovery rate (BH FDR)-corrected p-values were obtained. Taxa or genes were considered differentially abundant if the corrected p-value was less than 0.1 and if they were present in at least 50% of the samples in one of the compared groups. A Gene Set Enrichment Analysis (GSEA) was conducted using fgsea package in R version 1.16 (Korotkevich et al., 2019) on KEGG pathways. The log fold change of KEGG Orthology/annotated genes from the limma-voom differential abundance analysis was employed for the GSEA.

Data was normalized using rarefaction technique from Phyloseq R package (McMurdie and Holmes, 2013) in order to perform alpha-diversity analysis. Shannon, Simpson, and Richness indexes were calculated using vegan R package (Oksanen et al., 2016), and the Wilcoxon signed-rank test was used to find significant differences in alpha-diversity between groups.

Bray–Curtis dissimilarity matrix and Permutational analysis of variance (PERMANOVA) analysis for beta-diversity were performed using vegan R package, after normalization by relative frequency for each sample.

Spearman’s rank correlation was used to associate differentially abundant taxa with the hydrogen and methane levels at the phylum, family, genus, and species levels. Correlation coefficients and p-values were calculated using the Psych R package version 2.2.9. (Revelle, 2022) The corrplot function was applied to visualize the correlation matrices. Statistical significance was defined by a BH FDR adjusted p-value < 0.05.

#### 2.7.4 Analysis of fecal biomarkers of inflammation

Fecal samples were processed and analyzed using an enzyme linked immunosorbent assay (ELISA) to detect and quantify levels of the following biomarkers of inflammation: calprotectin, lactoferrin, and zonulin. ELISA kits for the three biomarkers were obtained from ImmunDiagnostik (Bensheim, Germany) and samples were processed and analyzed according to the manufacturer’s instructions.

Analyte concentrations were obtained from a 4-parameter logistic curve fitted to appropriate standard material. In all cases, standards, controls, and samples were analyzed in duplicate. Quality controls for each assay included 2 control materials of known concentrations and expected values were achieved in all cases when compared to the manufacturer’s quality control data. Fecal biomarkers of inflammation were analyzed using the Wilcoxon signed-rank test and p-values of < 0.05 were considered significant.

## 3 Results

### 3.1 Participant baseline demographics, compliance and adverse events reporting

A total of 41 healthy young adults were enrolled in this open-label study. The participants were all given the interventional product 14-strain probiotic blend. Two participants withdrew from the study; one contracted an infection that required treatment with antibiotics and ‘no reason’ for withdrawal was given by the other participant.

A summary of participants characteristics at baseline is shown in **Supplementary Table S1**. Twenty women and 21 men, aged 19 to 32 (mean [ ± SD] age: 24.6 [ ± 2.6] years) with an average BMI of 23.9 ( ± 2.6) kg/m^2^ took part in this trial. The studied cohort reported generally healthy lifestyles, 68% exercised more than 3 times a week and 73% were non-smokers. Forty three percent were pet owners and only one participant reported having a history of allergy (hay fever). Finally, 63% of participants did not take any antibiotics within the past two years.

At baseline, 65% of participants reported no experiences of abdominal pain, 73% reported having never or only occasionally experienced bloating and 54% of participants reported never or only occasional discomfort from gas. Abdominal pain was the least prominent of the reported GI discomforts and gas was comparatively more frequently experienced **Supplementary Table S1**.

There were no serious adverse events reported. There were a total of 11 adverse events (AE) reported during the 8 week probiotic consumption period. Of those four were likely related to product intake (flatulence, bloating, headache and nausea). The AE were mild and transient as resolved within 2 days.

Participants’ compliance with product consumption was modest with an average product intake of 83%. Only 73% of the participants took more than 80% of the given product.

The trial started in November 2019 in the UK. It should be noted that during the trial period, the UK experienced variable restrictions and lockdowns due to the COVID-19 pandemic. Compliance and missing data are partly explained by the challenges faced during that period.

### 3.2 Fecal taxonomic analysis

Shotgun metagenomic sequencing was used to assess structural and functional changes of the fecal microbiome before after supplementation with the multi-strain probiotic.

#### 3.2.1 Modest changes to the fecal microbiota in response to 14-strain probiotic intake

First, the global microbiome composition was investigated. The relative abundance of fecal bacteria in the healthy adult population at baseline is presented at the genus and species level in **Supplementary Figure S1**. At the end of the 56 days intervention period no significant differences in alpha diversity were observed and slight differences in beta diversity, compared to baseline (**Supplementary Figure S2**). Alpha diversity is a within sample measure of richness and evenness, whilst beta diversity is a between sample measure. Principal coordinate analysis (PCoA) based on Bray–Curtis was analyzed to obtain an initial evaluation of the clustering of the groups; however, no clustering at baseline or the end of intervention was detected (**Supplementary Figure S2C**). Probiotic intake did not elicit significant overall microbiota composition changes.

**Figure 2:**
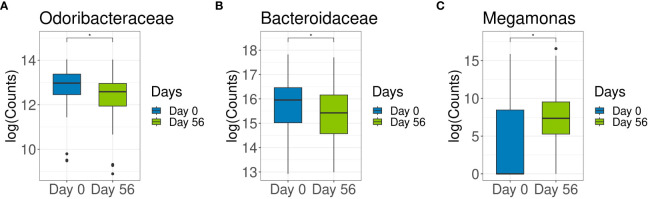
The effects of multi-strain probiotic intervention on abundance distribution of family *Odoribacteraceae* and *Bacteriodaceae* and the genus *Megamonas*. Abundance of *Odoribacteraceae* **(A)**, *Bacteriodaceae* **(B)** and *Megamonas* **(C)** in fecal samples collected at baseline (Day 0) and at the end of multi-strain probiotic intervention (Day 56 [ ± 2 days]) were compared by taxonomic analysis. (*) p < 0.05.

To elucidate the impact of specific covariates in the microbiome, we first tested the association of the microbial composition with the covariates of interest (sex, age, participant, and time) by PERMANOVA. PERMANOVA demonstrated a significant impact of sex, age, and individual participant on fecal bacteria metataxonomy, with individual participant having the highest impact (84%) (**Supplementary Tables S2, S3**). In other terms, age, sex, and individual participant were key variables that affected microbiota composition; however, the microbiota of each individual remained relatively stable through time (Day 0 to Day 56). In a simplified model, where only time and participant variables were included but sex and age variables were omitted a significantly lower abundance of families *Odoribacteraceae* (log2FoldChange = -0.54, p = 0.008) and *Bacteroidaceae* (log2FoldChange = -0.43, p = 0.03) were detected after 8 weeks of probiotic intervention (**Figures 2A, B**). When genera were analyzed, a significant increase in genus *Megamonas* (log2FoldChange = 5.06, p = 4.09E‐07) abundance was observed between baseline and end of probiotic intervention (**Figure 2C**).

Overall the 14-strain interventional product did not elicit significant microbiota compositional changes, however in a simplified model that included only time and participant variables, there was a significant reduction in abundance of *Odoribacteraceae* and *Bacteroidaceae* families, and increased abundance of *Megamonas* genus.

#### 3.2.2 Bacterial species present in the investigational product are detected in the fecal microbiome

**Figure 3:**
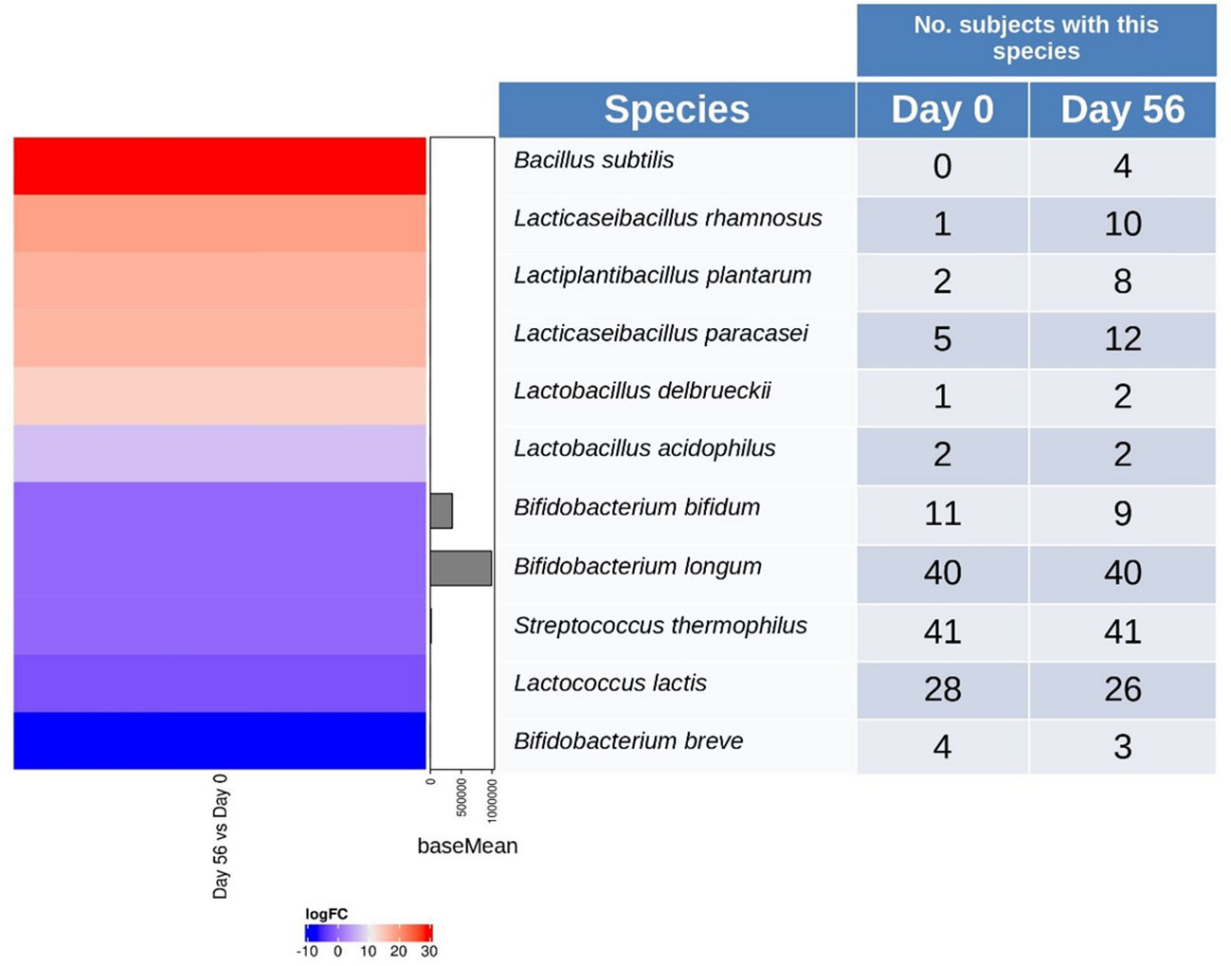
Heatmap and contingency table of detected multi-strain probiotic representatives. Left panel differential abundance species between baseline (Day 0) and end of multi-strain probiotic intervention on Day 56 ( ± 2 days). Species are shown over‐represented (red color) or under‐represented (blue color) in Day 56 respect to Day 0. Right panel, the number of participants in which the probiotic strains abundance was different from zero. Data was analyzed using the Wilcoxon signed-rank test and p-values of < 0.05 were considered significant.

The stool samples were analyzed to identify the species present in the multi-strain product. The samples were analyzed at baseline and at the end of the probiotic intervention period (**Figure 3**). At species level, *B. subtilis* (log2 FoldChange = 28.45, p = 1.41E-20), *L. rhamnosus* (log2 FoldChange = 18.6, p = 2.42E-09), *L. plantarum* (log2FoldChange = 16.89, p = 7.63E-08), and *L. paracasei* (log2FoldChange = 16.55, p = 1.42E‐07) were detected in more participants at the end of intervention, compared to baseline (adjusted p‐value < 0.05). *B. longum* and *S. thermophilus* was detected in the highest number of participants before and after probiotic intervention. A slight decrease in the number of participants presenting *B. bifidum* and *Lactococcus lactis* was observed. Overall, species from the 14-strain blend were differentially detected in the fecal microbiota of the participants after consumption of the probiotic.

### 3.3 Functional contribution of the interventional product on the fecal microbiome

Shotgun metagenomics data was used to assess microbiome functional changes in response to multi-strain probiotics intake.

**Figure 4:**
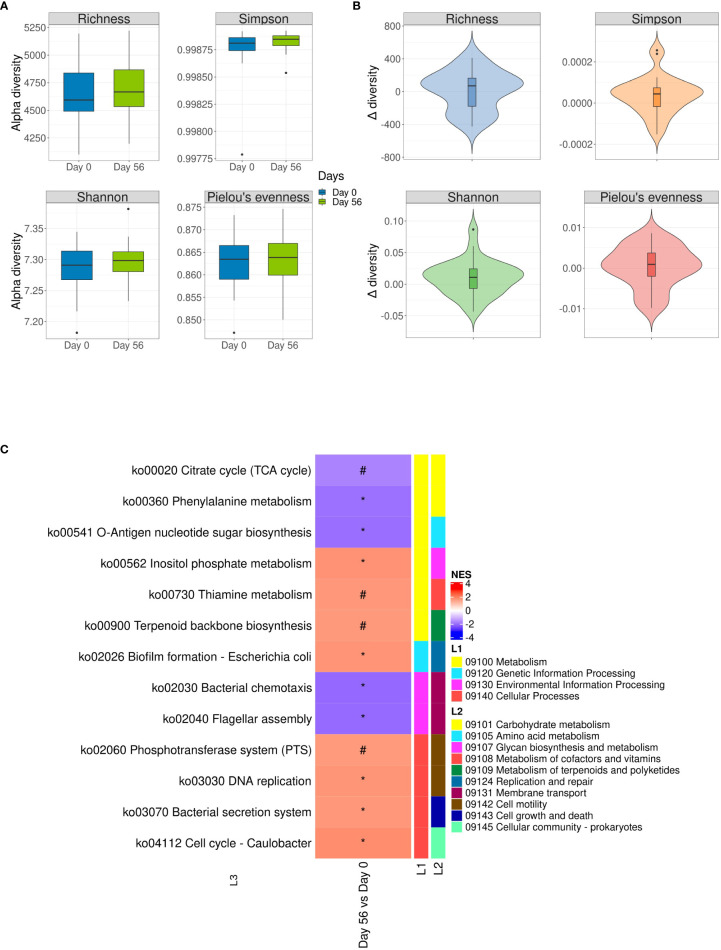
Functional alpha-diversity analysis expressed as values and as changes from baseline to the end of multi-strain probiotic intervention at species level. **(A)** Boxplots of four common alpha-diversity indices (Richness, Simpson, Shannon and Pielou’s evenness index) at baseline (Day 0) and end of multi-strain probiotic intervention on Day 56 ( ± 2 days). **(B)** Violin graph of delta alpha-diversity indexes (between Day 56 and Day 0 values) of the four alpha-diversity indices. Data was analyzed using the Wilcoxon signed-rank test and p-values of < 0.05 were considered significant. **(C)** Gene set enrichment analysis was performed to compare Kyoto Encyclopedia of Genes and Genomes pathways from fecal samples provided at baseline (Day 0) and end of multi-strain probiotic intervention on Day 56 ( ± 2 days). Pathways categories are shown over‐represented (red color) or underrepresented (blue color) in Day 56 respect to Day 0. (#) p > 0.1; (*): p < 0.05. L1: high-level functions and utilities; L2: General categories; L3: Pathway level. NES, Normalized Enrichment Scores.

Microbial functional alpha-diversity showed no change following multi-strain probiotics consumption (**Figure 4A**); although slight none significant positive delta values were observed when baseline was compared with the end of intervention (**Figure 4B**). PCoA based on Bray–Curtis (beta-diversity) was analyzed to obtain an initial evaluation of the functional clustering of the groups, with results displaying no clear clustering based on time (**Supplementary Figure S3**).

The Gene Set Enrichment Analysis (GSEA) was conducted to evaluate potential differential changes in function analysis between the KEGG groups in the hierarchical levels L1, L2 and L3 used by the KEGG classification (**Figure 4C**). At the end of intervention, enriched functions were included, but were not limited to inositol phosphate metabolism (normalized enrichment score [NES] = 1.67, p = 0.047), thiamine metabolism (NES = 1.59, p = 0.094), terpenoid neckbone biosynthesis (NES = 1.57, p = 0.07), phosphotransferase system (NES = 1.55, p = 0.05), DNA replication (NES = 1.62, p = 0.047), and bacterial secretion system (NES = 1.58, p = 0.047). The GSEA also rendered functions that were enriched at baseline compared with the end of intervention. These included phenylalanine metabolism (NES = ‐1.84, p = 0.010), O‐antigen nucleotide sugar biosynthesis (NES = -1.88, p = 0.011), bacterial chemotaxis (NES = -1.95, p = 0.010), and flagellar assembly (NES = -1.95 p = 0.010).

Although functional alpha-diversity did not change, gene set enrichment analysis showed statistically significant changes in certain functional gene abundance. These genes comprised functions involved in metabolism, genetic and environmental information processing and cellular processes.

### 3.4 Breath hydrogen and methane levels and taxonomic changes

**Figure 5:**
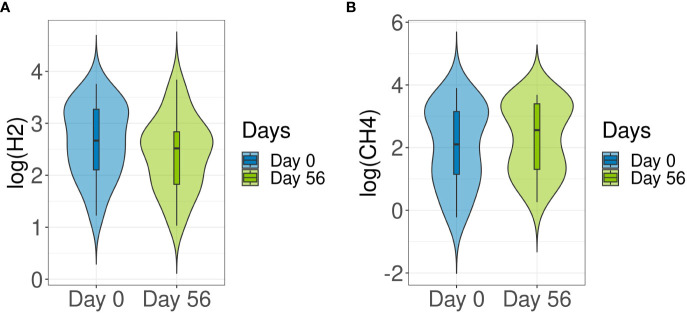
Comparison of hydrogen and methane production between baseline and end of multi-strain probiotic intervention. Participants were required to provide a fasted breath sample at baseline (Day 0) and during end of study visit (Day 56 ± 2 days). Hydrogen **(A)** and methane **(B)** values were determined from breath samples analyzed using gas chromatography to compare baseline (Day 0) and end of study (Day 56) readings. Data was analyzed using the Wilcoxon signed-rank test and p-values of <0.05 were considered significant. CH4, methane; H2, hydrogen.

Breath test analyses are used here to measure gas produced in the intestine. Hydrogen and methane are exclusively produced by bacterial fermentation in the gut. No significant changes in hydrogen (p = 0.589) or methane (p = 0.83) production compared to baseline were detected after probiotic intake (**Figure 5**). Following this, Spearman’s Rank correlation was applied to detect specific correlations between methane production and taxa. At phylum level, Euryarchaeota (R = 0.7, p = 5.27E-13) and Verrucomicrobia (R = 0.25, p = 0.02) were positively correlated with methane levels, whereas Firmicutes (R = -0.47, p = 1.03E-05), Bacteroidetes (R = -0.44, p = 3.8E-05) and Actinobacteria (R = -0.42, p = 9.25E-05), among others, were inversely correlated (**Supplementary Figure S4**). At family level, *Methanobacteriaceae* (R = 0.7, p = 3.46E-13) was found to be directly correlated with methane levels. At genus level, *Blautia* (R = -0.43, p = 1.2E-06), *Bacteroides* (R = -0.41, p = 3.98E-06), *Faecalibacterium* (R = -0.35, p = 0.0002) and *Bifidobacterium* (R = -0.22, p = 0.008), were inversely correlated with methane production, whereas *Methanobrevibacter* (R = 0.72, p = 6.05E‐14) and *Akkermansia* (R = 0.25, p = 0.07) were positively correlated (**Supplementary Figure S5A**). At species level, *Blautia wexlerae* (R = -0.44, p = 4.29E-05) and *F. prausnitzii* (R = -0.36, p = 0.0008) were inversely correlated with methane production, whereas *M. smithii* (R = 0.72, p = 5E-14) was positively correlated (**Supplementary Figure S5B**). The specific methane producers, Archaea *Methanobrevibacter* and its species *M. smithii*, were detected in 90% of samples at baseline and in 93% of samples after 8 weeks of multi-strain probiotic intervention (**Supplementary Figure S6**).

For analysis of specific correlations between hydrogen production and various taxa, at genus level, hydrogen levels were positively correlated with *Bifidobacterium* (R = 0.35, p = 0.002) and *Hydrogeniiclostridium* (R = 0.3, p = 0.008), among others (**Supplementary Figure S7A**). At species level, *Bacteroides ovatus* (R = 0.32, p = 0.004), *B. adolescentis* (R = 0.32, p = 0.003) and *B. salyersiae* (R = 0.3, p = 0.007), were positively correlated with hydrogen production and only *Slackia isoflavoniconvertens* (R = -0.33, p = 0.003) was inversely correlated (**Supplementary Figure S7B**).

Breath test analysis showed no changes in methane and hydrogen production levels after probiotic intake in this healthy adult cohort. Correlation analysis linked certain family, genus and species with hydrogen and methane production.

### 3.5 Fecal biochemistry analysis

There were no significant changes in fecal levels of zonulin (p = 0.387), calprotectin (p = 0.676), or lactoferrin (p = 0.766) between baseline and end of multi-strain probiotic intervention. (**Supplementary Figure S8**).

### 3.6 Stool frequency and consistency

Bowel habits and stool form were recorded by participants in study diaries. These parameters were assessed at week 1, week 5 and week 8 in healthy young adults. Week 1 was considered as baseline. Compared with baseline, there were no significant changes in stool frequency (p=0.813) following 8 weeks of multi-strain probiotic intervention (**Supplementary Figure S9**). The healthy cohort had on average 1.47 bowel movements a day, this was slightly increased to 1.64 bowel movements a day at week 5 however this did not reach significance (p=0.293). This healthy adult population maintained a consistent bowel movement frequency throughout the probiotic intervention period. In terms of stool consistency no significant changes were observed between week 1 and week 8 (Chi-square=4.07, p=0.396) (**Supplementary Figure S10**). At week 1, 70% of participants reported healthy stool form, 2.5% reported diarrhea and 27.5% reported being constipated. At week 5, 86.11% of participants reported having healthy stool but this improvement from baseline did not reach statistical significance. At week 8 there were slightly more participants (75.76%) reporting healthy stool form compared to week 1 but again this did not reach statistical significance. Overall, during the 8-week probiotic intervention period no significant changes were observed for defecation frequency and stool consistency.

## 4 Discussion

To our knowledge this is the first clinical trial to evaluate structural and functional fecal microbiome response to this 14-strain probiotic intake by shotgun metagenomics in healthy individuals. The results presented here showed that alpha- and beta-diversity of the fecal microbiota structure was not significantly altered in response to probiotic intake. However, significant changes were observed when functional genes were assessed. Abundance of certain genes involved in several functional pathways, including phenylalanine metabolism, O-antigen nucleotide sugar biosynthesis, bacterial chemotaxis, and flagellar assembly were significantly altered following probiotic intake. No significant changes in stool frequency or consistency, fecal biochemistry, or breath tests of methane and hydrogen were observed.

It has been widely observed that, in healthy individuals, the alpha- and beta diversity of gut microbiome composition tend to be stable in response to probiotic intervention. A systematic review conducted in 2016 by Kristensen et al. assessed seven RCTs investigating the effect of probiotic supplementation on the gut microbiota of healthy adults and concluded that probiotic supplementation did not affect composition in healthy adults (Kristensen et al., 2016). The results of our single-arm probiotic intervention in healthy adult are consistent with these previous studies.

Despite the stability of the overall microbial composition among healthy adults, probiotic supplementation has been shown to support many aspects of health and well-being. Previous studies based on metabolic pathway analysis have reported a dissociation between microbiota structure and function, demonstrating that functionality can change in response to dietary intervention with minimal compositional changes (O’Keefe et al., 2015). The functional changes to the fecal microbiome observed in this exploratory study offers new perspective on how probiotics might support aspects of health without disturbing microbiome structure in healthy adults. Functional data from this study might even open a debate on the importance of product viability through the GI tract and supports the idea that in a healthy population probiotics might elicit beneficial effect through other routes than viability and subsequent colonization of the gut. Indeed, it would be interesting to look at functional changes to the healthy gut microbiome in response to postbiotics to understand if a similar effect is seen. Future studies are needed to understand the effect of these additional functions on host health.

Another interesting result from this trial is the product specific species analysis of the fecal sample. The data showed that the number of individuals presenting with product specific species after 8 weeks of intervention depended on the species and individual. The main limitation to consider here was that the data was not analyzed at strain level. The differential contribution of product specific probiotic strains on host health is an interesting angle that should be explored further. This though is a hugely complex task given the complex bacterial consortia that makes up the multi-strain probiotic product. It should be noted that some product specific species had a lower prevalence (bacteria present in less than 50% of the samples) in the fecal microbiome of health adults, this should be further assessed.

There were no serious adverse events reported and only few mild and transient adverse events recorded. Consistent with other studies, the consumption of multi strain probiotics at a dose of 8x10^9^ CFU/day in healthy adult participants had no significant effect on bowl habits, stool consistency or fecal inflammatory markers. A number of issues blur the conclusions that can be drawn from this studies, including small sample sizes, lack of a control group, compliance issues, inter-individual variation in susceptibility toward the probiotic, duration of intervention and lack of information on participants diet.

Despite these limitations the data provided here in terms of composition, diversity, and functional gene alterations in fecal microbiome of healthy individuals offer an important foundation for our understanding of intestinal microbiome dynamics in response to multi-strain probiotic intake.

## 5 Conclusion

In summary, using a metagenomics approach, this study demonstrated changes in abundance of functional genes following 8 weeks of multi-strain probiotic consumption. No significant changes were observed in stool form or frequency, fecal biochemistry, or methane and hydrogen breath tests. This lack of change was expected and attributed to the study being carried out in a healthy population. Results from this study have the potential to provide insights into the underlying mechanisms of action of the 14 strain probiotic blend in healthy adults. This to our knowledge has never been done before and this open label study is a stepping stone many future research to come. Understanding how functional alterations in microbial genes and subsequent metabolic pathway alteration can contribute to improve our knowledge in this rapidly evolving area of research.

## Data availability statement

The datasets presented in this study can be found in online repositories. The names of the repository/repositories and accession number(s) can be found below: Sequence Read Archive (SRA) submission: SUB13715721. BioProject: PRJNA1002850.

## Ethics statement

The studies involving humans were approved by South West - Central Bristol Research Ethics Committee Whitefriars. The studies were conducted in accordance with the local legislation and institutional requirements. The participants provided their written informed consent to participate in this study.

## Author contributions

SB, AH, ST, JH, MN, and RD designed the clinical study. SB, AH, ST, and JH conducted the clinical study and VV, MN, and RD supervised the analysis. AL and EC designed the gut microbiota study. AR-G, AL performed the bioinformatic and statistical analysis and EC supervised the analysis. SB, AH, ST, JH, VV, AR-G, AL wrote the first draft with inputs from EC, VV, MN, and RD. All authors were involved in data interpretation and discussion of the results. All authors contributed to the article and approved the submitted version.
