# Supplementary material for: An insight into the functional alterations in the gut microbiome of healthy adults in response to a multi-strain probiotic intake: a single arm open label trial
Source: Front Cell Infect Microbiol. 2023 Sep 29;13:1240267. doi: 10.3389/fcimb.2023.1240267 (PMC10570534; doi:10.3389/fcimb.2023.1240267)
Supplement: Supplementary file 1 [file DataSheet_1.docx]

## **[Table S1. Participant Disposition](#_Table_S1_–) and Baseline Demographics**

|  | Participants |
| --- | --- |
| Sex |  |
| Male; n (%) | 21 (51.2) |
| Female; n (%) | 20 (48.8) |
| Age in years (mean ± SD) | **24.61 ± 2.60** |
| BMI (mean ± SD) (kg/m^2^) | **23.87 ± 2.62** |
| Exercise; n (%) |  |
| <3 times a week | 8 (20) |
| 3–5 times a week | 13 (32) |
| >5 times a week | 15 (37) |
| Smoking; n (%) |  |
| No | 30 (73) |
| Occasionally | 6 (15) |
| Yes | 1 (2) |
| Owns a Pet; n (%) |  |
| Yes | 18 (44) |
| No | 19 (46) |
| Missing Data | 4 (10) |
| Allergies and Intolerances; n (%) |  |
| No | 36 (88) |
| Yes | 1 (2) |
| Missing Data | 4 (10) |
| Antibiotic Use Within the Past 2 Years; n (%) |  |
| No | 26 (63) |
| Yes |  |
| 1 Course | 8 (20) |
| 2 Courses | 4 (10) |
| 3+ Courses | 2 (5) |
| Missing Data | 1 (2) |
| Education |  |
| High School | 8 (20) |
| Undergraduate Degree | 21 (51) |
| Graduate Degree | 4 (10) |
| Associate Degree | 5 (12) |
| Other | 3 (7) |
| Baseline GI Symptoms: |  |
| Incidence of Gas; n (%) |  |
| Never | 5 (12) |
| Occasionally | 17 (41) |
| Frequently | 11 (27) |
| Usually | 3 (7) |
| All the time | 1 (2) |
| Missing Data | 4 (10) |
| Incidence of Bloating; n (%) |  |
| Never | 15 (37) |
| Occasionally | 15 (37) |
| Frequently | 4 (10) |
| Usually | 3 (7) |
| All the time | 0 (0) |
| Missing Data | 4 (10) |
| Incidence of Abdominal Pain; n (%) |  |
| Never | 27 (66) |
| Occasionally | 8 (20) |
| Frequently | 5 (12) |
| Usually | 0 (0) |
| All the time | 0 (0) |
| Missing Data | 1 (2) |

BMI, body mass index; GI, gastrointestinal; n, number of participants; SD, standard deviation.

## [**Table S2.**](#_Table_S2._Permutational) **Permutational Analysis of Aariance Analysis in Metataxonomy**

| Parameter | Df | SumOfSqs | R2 | F | Pr(>F) |
| --- | --- | --- | --- | --- | --- |
| Time | 1 | 0.0398 | 0.00254 | 0.8531 | 0.672 |
| Sex | 1 | 0.4016 | 0.02561 | 8.6108 | 0.001*** |
| Age | 1 | 0.2042 | 0.01302 | 4.3774 | 0.001*** |
| Participant | 38 | 13.1718 | 0.83987 | 7.4313 | 0.001*** |
| Residual | 40 | 1.8658 | 0.11897 | – | – |
| Total | 81 | 15.6832 | 1 | – | – |

Df, Degrees of freedom; F, F-statistic; Pr(>F), P-value for F-statistics.; R2, R-squared or coefficient of determination

## **Table S3. Permutational Analysis of Variance Analysis in Functional Study**

| Parameter | Df | SumOfSqs | R2 | F | Pr(>F) |
| --- | --- | --- | --- | --- | --- |
| Time | 1 | 0.00483 | 0.00803 | 1.5562 | 0.091 |
| Sex | 1 | 0.01399 | 0.02325 | 4.5080 | 0.001*** |
| Age | 1 | 0.00759 | 0.01262 | 2.4476 | 0.009** |
| Subject | 37 | 0.49139 | 0.81683 | 4.2800 | 0.001*** |
| Residual | 27 | 0.08378 | 0.13927 | – | – |
| Total | 67 | 0.60159 | 1.00000 | – | – |

Df, Degrees of freedom; F, F-statistic; Pr(>F), P-value for F-statistics.; R2, R-squared or coefficient of determination


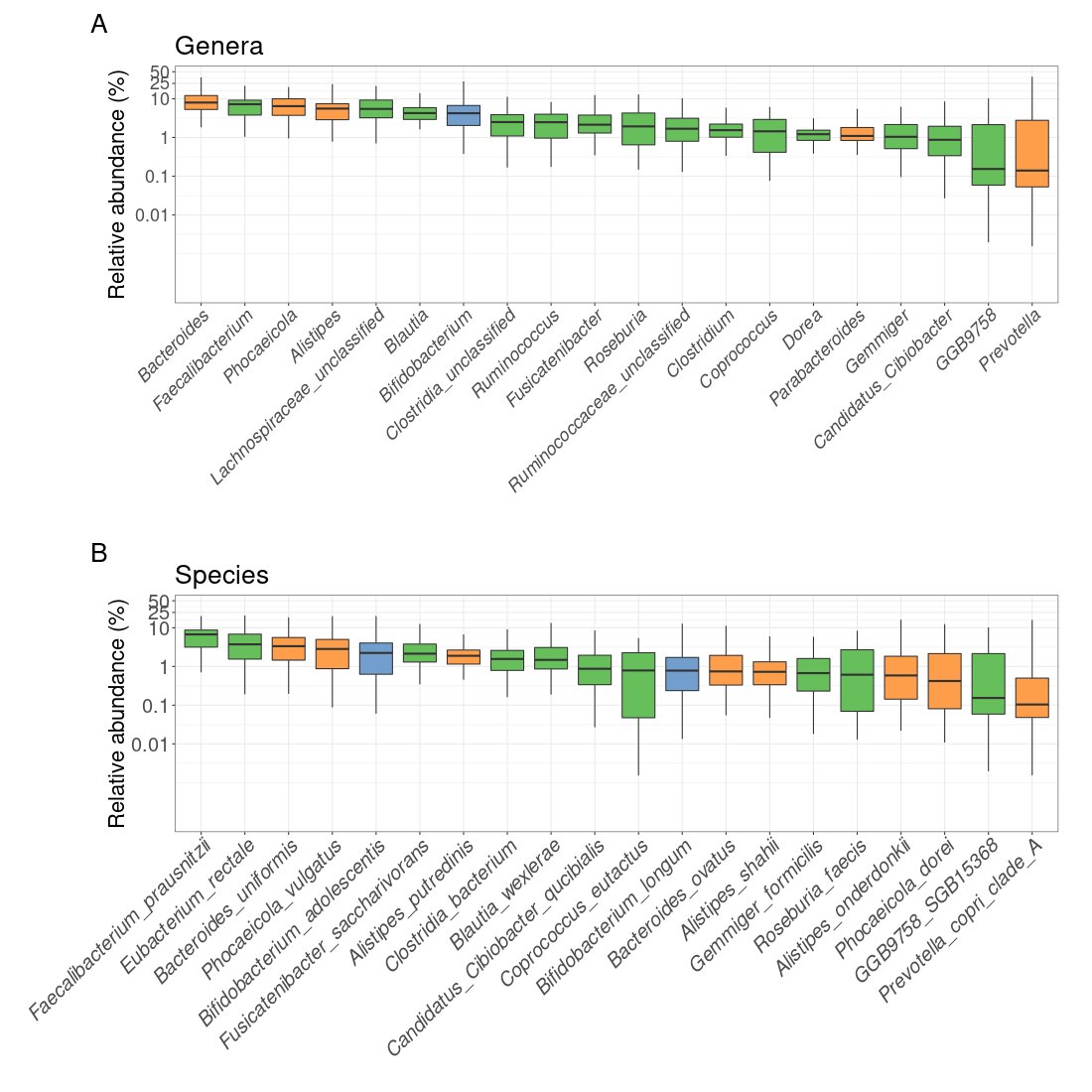


## **Figure S1: Box Plots of Relative Abundance of Top 20 Taxa in the Population at Day 0.** A) genera B) species. Blue: Actinobacterium phylum, Orange: Bacteroidetes phylum, Green: Firmicutes phylum.


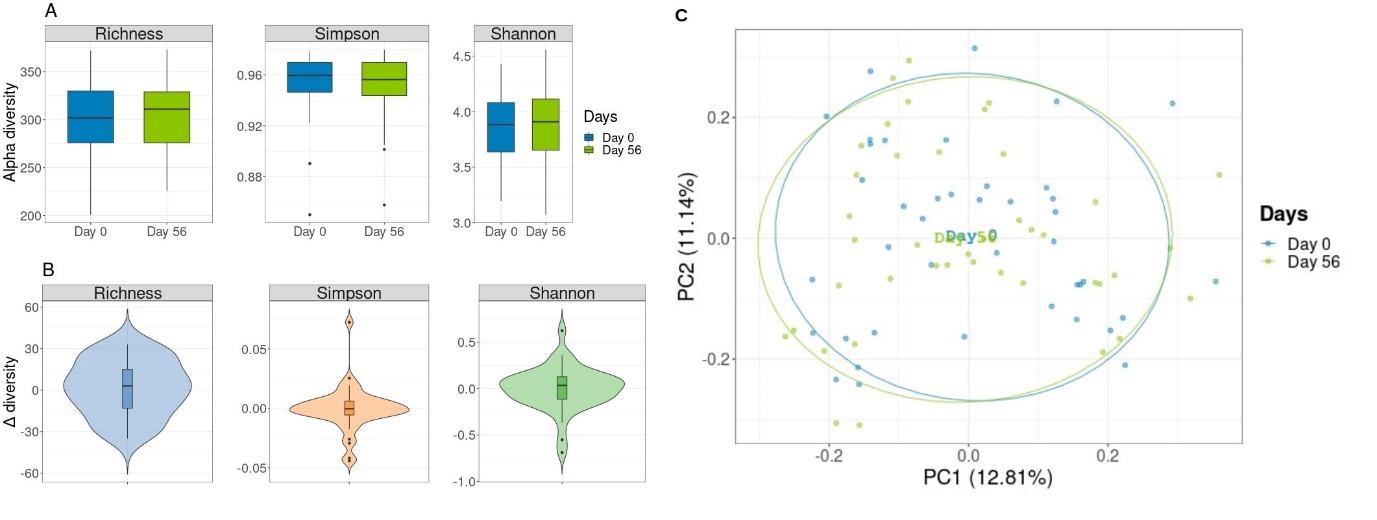


## **Figure S2: Richness and Alpha-Diversity Values Over Time and Difference Between Baseline and End of multi-strain probiotic Intervention.** A) Boxplots of three common alpha‑diversity indices (Richness, Simpson, Shannon index) at baseline (Day 0) and end of multi-strain probiotic intervention on Day 56 (±2 days). B) Violin graph of delta alpha-diversity indexes (between Day 56 and Day 0 values) of the three alpha-diversity indices. Data was analyzed using the Wilcoxon signed‑rank test and p-values of < 0.05 were considered significant. C) PCoA using the Bray–Curtis distance measures at baseline (Day 0) and end of multi-strain probiotic intervention on Day 56 (±2 days), based on taxonomic profiles of the samples. The points were colored according to the time.

PCoA, Principal coordinate analysis.


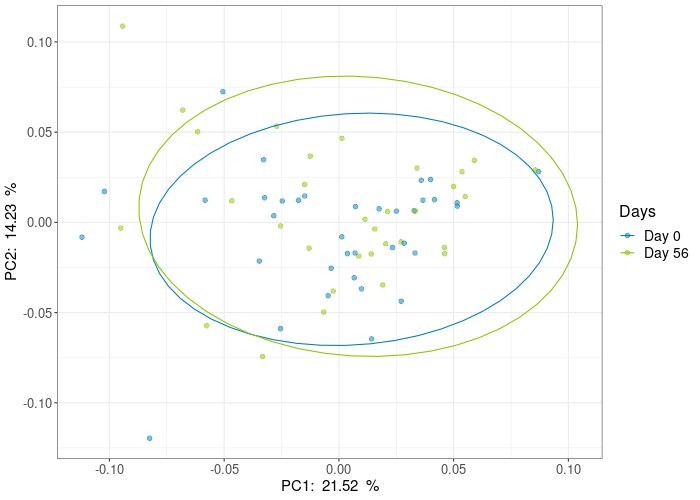


**Figure S3: Functional Principal Coordinate Analysis of Beta-Diversity at Baseline and the End of multi-strain probiotic Intervention.** PCoA using the Bray–Curtis distance measures at baseline (Day 0) and end of multi-strain probiotic intervention on Day 56 (±2 days), based on functional profiles of the samples. The points were colored according to the time.

PCoA, Principal coordinate analysis.


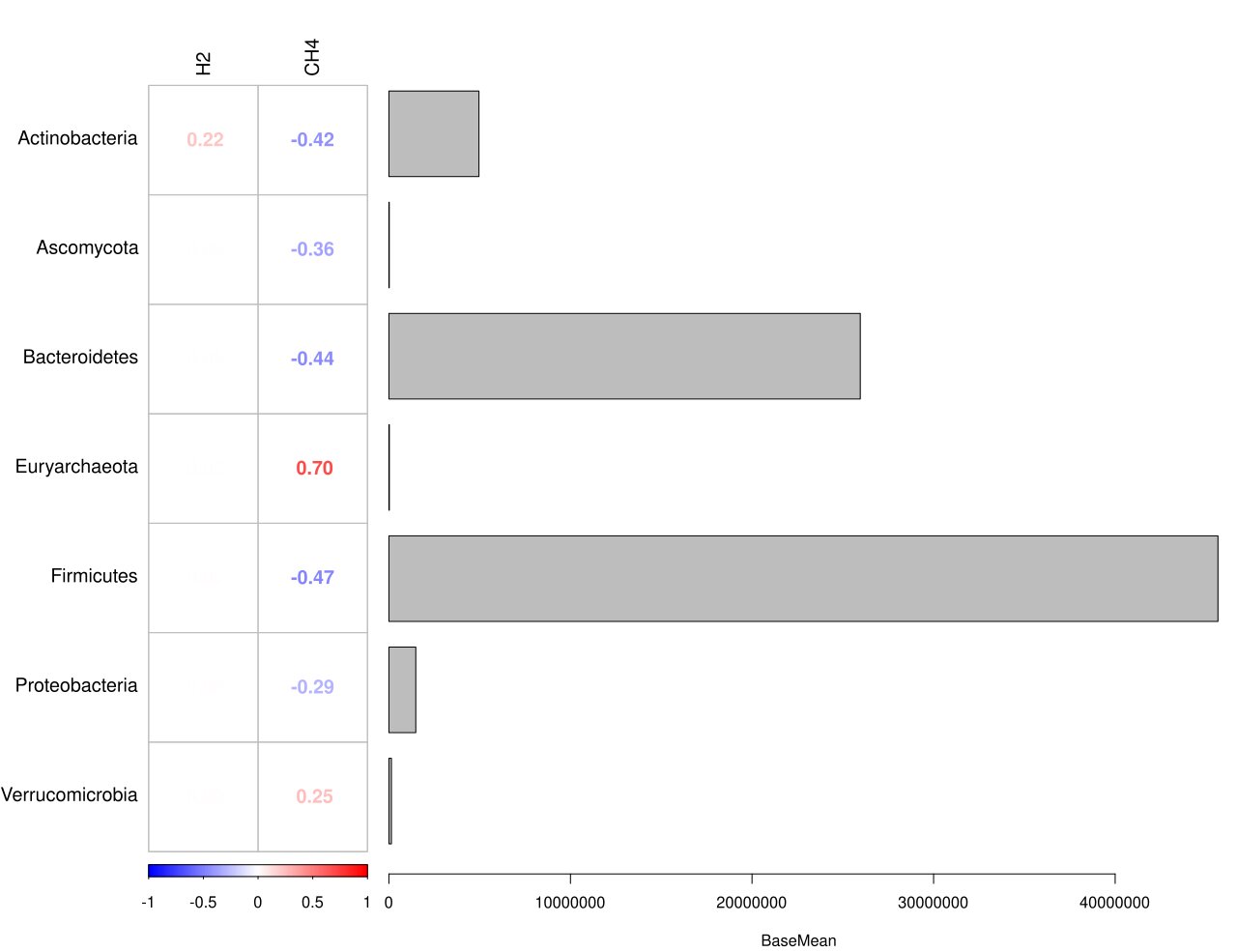


**Figure S4: Spearman’s Correlation Between Hydrogen and Methane and Taxa at Phylum Level** The numbers in the boxes indicate Spearman's rank correlation coefficient. Red numbers: positive correlation. Blue numbers: negative correlation. The Bar plot represents the mean abundance of the taxa in all the samples.

CH4, methane; H2, hydrogen.


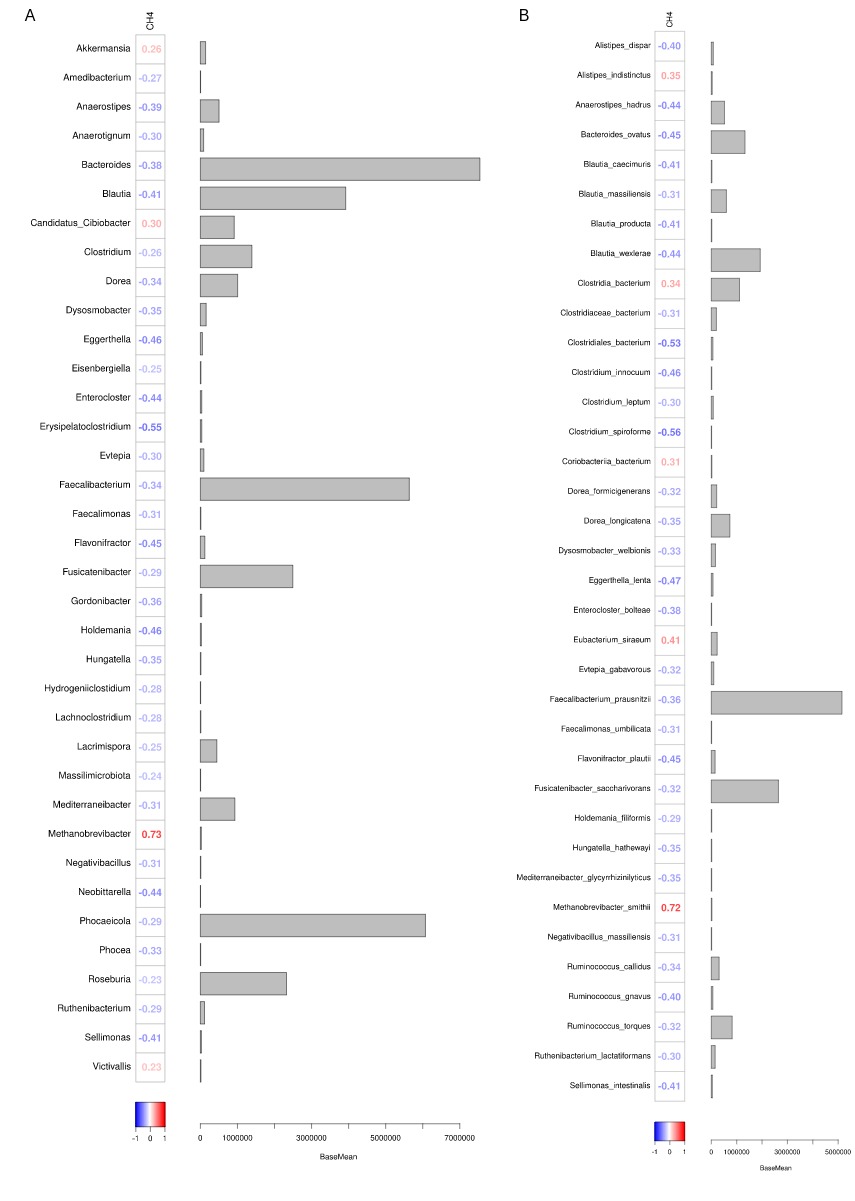


**Figure S5: Spearman’s Correlation Between Methane and Taxa at A) Genus Level and B) Species Level.**
The number in the boxes indicate Spearman's rank correlation coefficient. Red numbers: positive correlation. Blue numbers: negative correlation. The Bar plot represents the mean abundance of the taxa in all the samples.

CH4, methane.


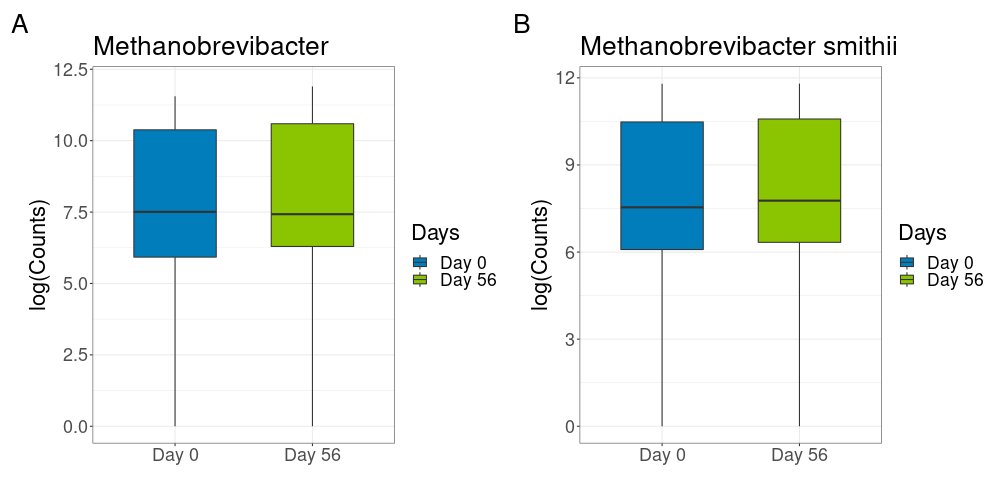


**Figure S6: Analysis of Methane Producers Methanobrevibacter and M. smithii.** Abundance of *Methanobrevibacter* and *M. smithii* in fecal samples collected at baseline (Day 0) and at the end of multi-strain probiotic intervention (Day 56 [±2 days]) were compared by taxonomic analysis. Data was analyzed using the Wilcoxon signed-rank test and p-values of < 0.05 were considered significant.


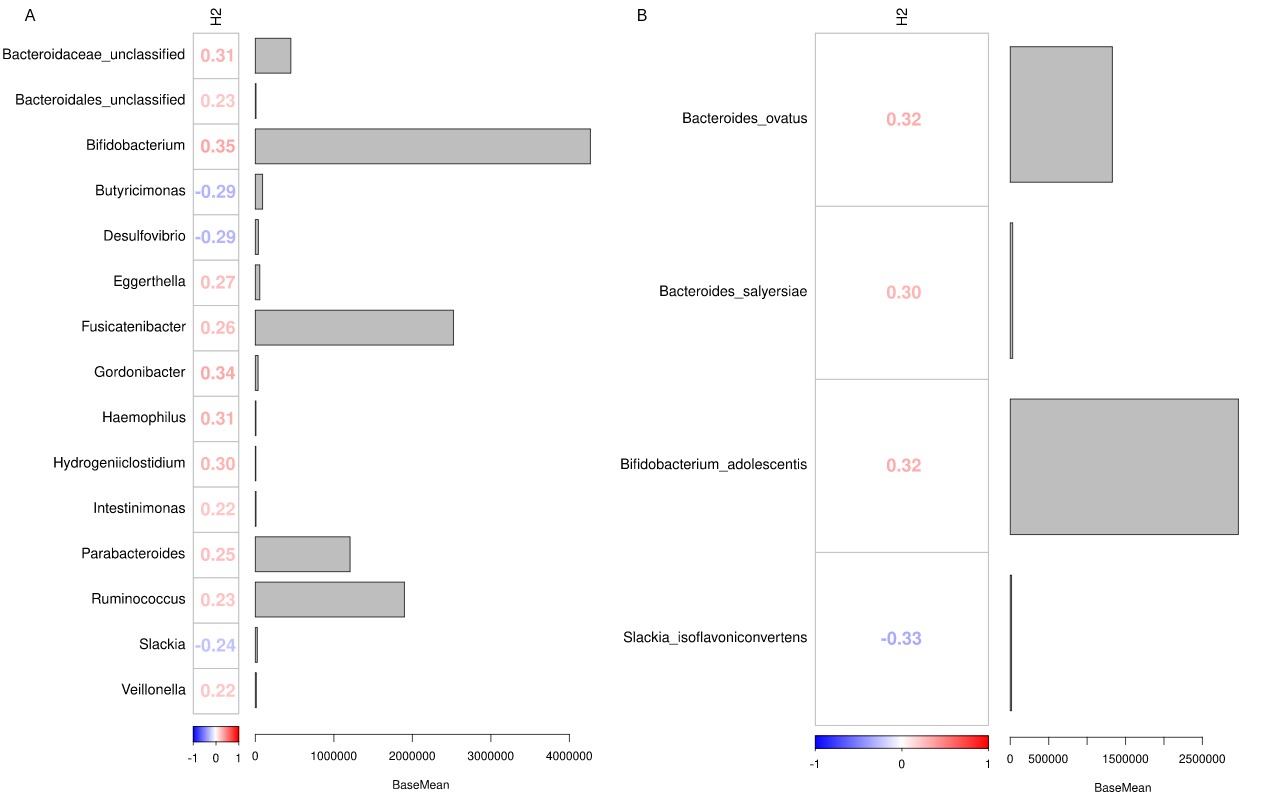


**Figure S7: Spearman’s Correlation Between Hydrogen and Taxa at A) Genus Level and B) Species Level.**
The numbers in the boxes indicate Spearman's rank correlation coefficient. Red number: positive correlation. Blue number: negative correlation. The Bar plot represents the mean abundance of the taxa in all the samples.

H2, hydrogen.


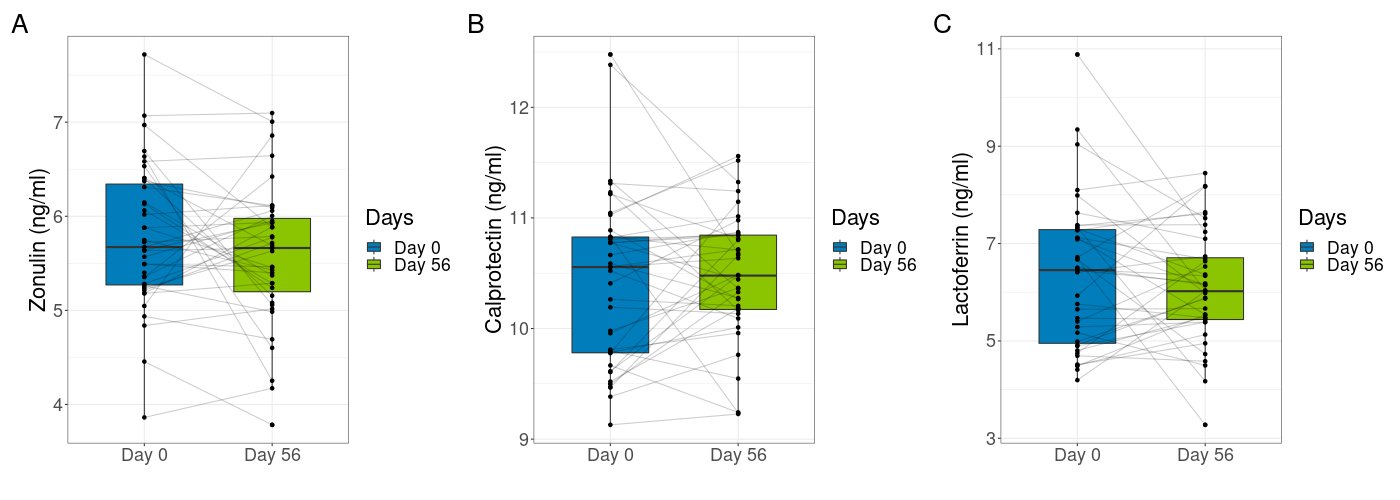


**Figure S8: Zonulin, Calprotectin, and Lactoferrin Levels at Baseline and the End of multi-strain probiotic Intervention.** The quantity of Zonulin, Calprotectin and Lactoferrin from 80 fecal samples were determined by ELISA and comparisons between baseline (Day 0) and post multi-strain probiotic intervention (Day 56 [±2 days]) were performed. Data was analyzed using the Wilcoxon signed-rank test and p-values of < 0.05 were considered significant.

**
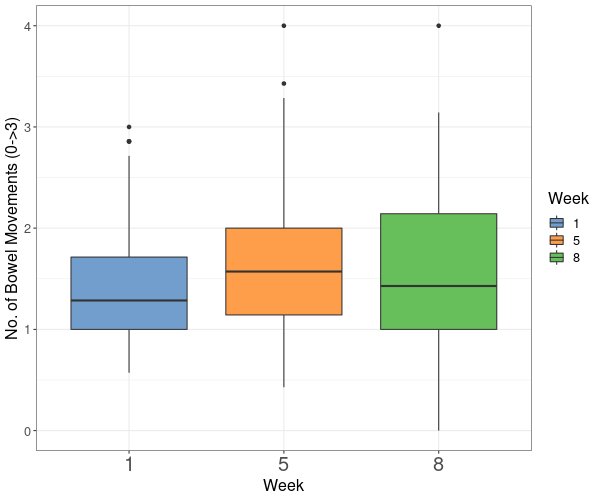
**

**Figure S9: Bowel Movements of Study Participants.** The number of bowel movements recorded in study diaries between baseline and end of study were recorded and are presented at 1, 5, and 8 weeks after multi-strain probiotic intervention. Paired t-tests were performed between the number of bowel movements at weeks 1, 5, and 8. P-values of < 0.05 were considered significant.

**
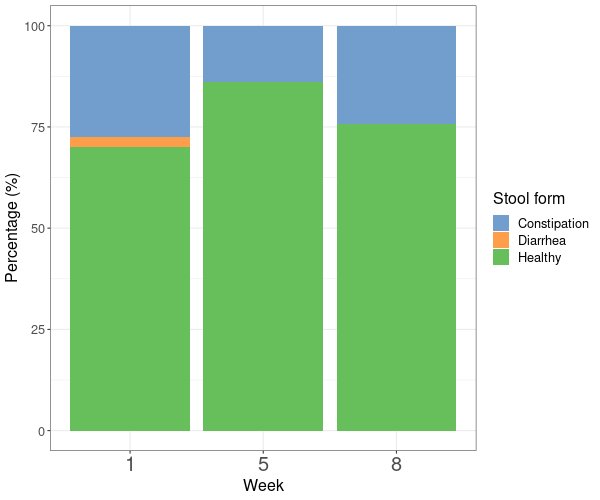
**

**Figure S10: Stool Form of Participants Over 8 Weeks of multi-strain probiotic intake.** The percentage of individuals with constipation, diarrhea, and healthy stool forms at baseline (Day 0) and at the end of multi-strain probiotic intervention (Day 56 [±2 days]). Data was analyzed using Chi-square and p‑values of < 0.05 were considered significant.
